# Plasma Interleukin-35 Levels Predict the Prognosis in Patients with HBV-Related Acute-on-Chronic Liver Failure

**DOI:** 10.3390/v16121960

**Published:** 2024-12-20

**Authors:** Liujuan Ji, Xue Mei, Wei Yuan, Hongying Guo, Yuyi Zhang, Zhengguo Zhang, Ying Zou, Yu Liu, Hui Zhu, Zhiping Qian, Yinzhong Shen

**Affiliations:** 1 Department of Liver Intensive Care Unit, Shanghai Public Health Clinical Center, Fudan University, Shanghai 201508, China; jiliujuan@shphc.org.cn (L.J.); meixue@shphc.org.cn (X.M.); yuanwei@shphc.org.cn (W.Y.); guohongying@shphc.org.cn (H.G.); zhangyuyi@shphc.org.cn (Y.Z.); zhangzhengguo@shphc.org.cn (Z.Z.); zouying@shphc.org.cn (Y.Z.); liuyu@shphc.org.cn (Y.L.); zhuhui@shphc.org.cn (H.Z.); 2 Department of Infection and Immunity, Shanghai Public Health Clinical Center, Fudan University, Shanghai 201508, China

**Keywords:** interleukin-35, hepatitis B virus, acute-on-chronic liver failure, prognosis

## Abstract

This study aimed to investigate the impact of IL-35 on the prognosis of patients with HBV-ACLF. We recruited 69 patients with HBV-ACLF, 20 patients with chronic hepatitis B (CHB), 17 patients with liver cirrhosis (LC), and 20 healthy controls (HCs) from a regional infectious disease treatment center in China. Plasma levels of IL-35 at baseline were detected using ELISA. Plasma IL-35 levels in the HBV-ACLF group were the highest among all four groups. Furthermore, survivors exhibited significantly higher IL-35 levels than non-survivors (*p* < 0.001). IL-35 levels correlated with MELD (r = −0.678, *p* < 0.001), COSSH-ACLF IIs (r = −0.581, *p* < 0.001), alpha-fetoprotein (AFP) (r = 0.433, *p* < 0.001), creatinine (Cr) (r =−0.396, *p* = 0.001), and lactate (r =−0.38, *p* =0.001). The combination of plasma IL-35 and MELD score had the highest mortality prediction efficiency, with an area under the curve (AUC) of 0.895 (95% CI: 0.812–0.978, *p* < 0.001), a sensitivity of 80.6%, and a specificity of 93.9%. Additionally, the Kaplan–Meier analysis revealed that lower levels of IL-35 (≤191.5pg/mL) were associated with poorer survival rates in HBV-ACLF patients (*p* < 0.001). Our results demonstrated that IL-35 could be an effective predictive marker for the prognosis of HBV-ACLF and improve the predictive performance when combined with the MELD score.

## 1. Introduction

Acute-on-chronic liver failure (ACLF) is characterized by the rapid deterioration of pre-existing chronic liver disease, often accompanied by systemic inflammation, multi-system organ failure, and extremely high short-term mortality [1,2]. To date, more than 250 million individuals are chronically infected with hepatitis B virus (HBV) worldwide [3]. HBV-related ACLF (HBV-ACLF) is the primary type of ACLF in China and many other Asian countries, with a mortality rate as high as 50–90% [4]. Liver transplantation remains the most effective treatment strategy, but it is usually limited by a shortage of donor livers. Therefore, accurate and early prediction for identifying patients with potentially high mortality is essential for the effective management of HBV-ACLF.

Several predictive scoring models have been developed for the prognosis of ACLF, including the model for end-stage liver disease (MELD), CLIF Consortium ACLF score (CLIF-C ACLFs), Chronic Liver Failure-Sequential Organ Failure Assessment (CLIF-SOFA), and Chinese Group on the Study of Severe Hepatitis B ACLF II score (COSSH-ACLF IIs) [4,5,6,7]. However, most of the models were developed in Western countries, where the main causes of disease are drugs and alcohol rather than HBV. The effectiveness of these scoring systems in predicting the prognosis of HBV-ACLF remains questionable. The COSSH-ACLF IIs, a prognostic score recently developed by a team in China, has demonstrated excellent predictive ability for HBV-ACLF. However, the scoring items are complex and fail to adequately account for the immune–inflammatory profile. Thus, a reliable and convenient biomarker is urgently needed to predict the short-term prognosis of patients in the early stage of HBV-ACLF, which is beneficial for timely identifying high-risk patients, delivering effective treatment strategies, and reducing mortality [5,8].

It is widely known that systemic inflammation and altered immune status play crucial roles in the progression and mortality of ACLF [9,10]. The interleukin-35 (IL-35), a newly identified member of the interleukin-12 (IL-12) family, is an anti-inflammatory and immunosuppressive cytokine primarily secreted by regulatory T cells (Tregs) and regulatory B cells (Bregs) [11]. As a heterodimeric cytokine composed of an α chain (p35 subunit) and a β chain (Epstein–Barr virus-induced gene 3, EBI3) [12], IL-35 binds to its receptors, which are homodimers or heterodimers composed of interleukin 12 receptor β2 (IL-12Rβ2) and glycoprotein 130 (GP130). This binding activates the JAK-STAT (Janus kinase/signal transducer and activator of transcription) pathway to initiate transcription of downstream genes [13,14]. IL-35 plays an important role in infectious diseases, autoimmune diseases, and cancer progression [14,15,16]. Numerous studies have demonstrated that IL-35 exerts a protective role in liver injury by mitigating inflammation, apoptosis, and fibrotic reactions [15,17,18]. Moreover, a previous study found that serum IL-35 levels were significantly higher in patients with prophase liver failure (PLF) compared with healthy controls and those with chronic viral hepatitis, and elevated IL-35 levels were associated with better prognosis in patients with PLF [19]. However, the levels of IL-35 in HBV-ACLF and their prognostic significance have not been thoroughly elucidated. Therefore, this study aimed to explore whether IL-35 levels in patients with HBV-ACLF correlate with their prognosis.

## 2. Methods

### 2.1. Study Subjects

A total of 126 subjects (HBV-ACLF, n = 69; chronic hepatitis B, CHB, n = 20; liver cirrhosis, LC, n = 17; healthy controls, HCs, n = 20) were prospectively recruited from Shanghai Public Health Clinical Center, Fudan University, Shanghai, China (from September 2021 to March 2024). All participants or their guardians provided informed consent. This study was performed according to the principles of the Helsinki Declaration II and was approved by the Human Ethics Committees of the Shanghai Public Health Clinical Center of Fudan University (approval No. 2024-S071-02).

### 2.2. Disease Definition and Outcome

HBV-ACLF was diagnosed according to the COSSH-ACLF criteria [4]: pre-existing CHB with or without evidence of cirrhosis; total bilirubin (TBil) ≥ 12 mg/dL; international normalized ratio (INR) ≥ 1.5 or prothrombin activity (PTA) ≤ 40%. Exclusionary criteria: 1. age ≥ 80 or ≤18 years old; 2. pregnancy; 3. co-infection with other hepatitis viruses (A, C, D, or E), cytomegalovirus (CMV), Epstein–Barr virus (EBV), and human immunodeficiency virus (HIV); 4. combined with other causes of hepatitis; 5. presence of other chronic non-hepatic diseases, such as diabetes, hypertension, kidney diseases, hematological diseases, or hyperthyroidism; 6. hepatocellular carcinoma and other tumors; 7. duplicate cases or patients with lost clinical data. COSSH-ACLF comprises three grades: ACLF-1, ACLF-2 and ACLF-3. ACLF-2 and ACLF-3 were combined into ACLF-2/3 due to the limited sample size of ACLF-3 in this study.

ACLF-1 includes four subgroups: 1. patients with kidney failure alone; 2. patients with liver failure alone with an INR ≥ 1.5 and/or kidney dysfunction and/or hepatic encephalopathy (HE) grade I or II; 3. patients with a single organ failure in the coagulation, circulatory, or respiratory system, and/or kidney dysfunction and/or HE grade I or II; 4. patients with cerebral failure plus kidney dysfunction. ACLF patients with 2 or ≥3 organ failures are defined as ACLF-2 and ACLF-3, respectively.

All HBV-ACLF patients were followed for at least 180 days to assess their long-term clinical outcomes. Based on the 180-day outcomes, patients with HBV-ACLF were further divided into survival (ACLF-S subgroup, n = 33)—characterized by significantly improved clinical symptoms, physical signs, and liver function (TBIL < 5 times the normal upper limit, PTA > 40%, or INR < 1.5) and non-survival individuals (ACLF-NS subgroup, n = 36), including those who died or underwent living-donor liver transplantation.

CHB patients were diagnosed based on their HBsAg results and/or HBV DNA positivity for more than 6 months [20]. LC was defined as stable, compensated cirrhosis, and patients were diagnosed based on one or more of the following criteria: pathology; endoscopy (esophageal and gastric varices); radiology (portal hypertension and/or liver nodularity); laboratory examination; and clinical evidence of previous decompensation [21]. Patients with a history of decompensation, including ascites, hepatic encephalopathy (HE), upper gastrointestinal hemorrhage, or infection were excluded. Healthy controls are participants with normal physical examination results.

### 2.3. Determination of Plasma IL-35 Levels

Blood samples were collected upon admission and stored at −80 °C. Plasma IL-35 levels were measured using a human IL-35 ELISA kit (Novus Biologicals, Toronto, ON, Canada) according to the manufacturer’s protocol. The detection range of IL-35 spanned from 15.6 to 1000 pg/mL.

### 2.4. Statistical Analysis

Statistical analyses were performed using SPSS 25.0 software (SPSS Inc., Chicago, IL, USA), and figures were created using GraphPad Prism 9.0 (GraphPad Software Inc., La Jolla, CA, USA). Continuous variables were presented as the mean ± standard deviation (SD) or medians (p25, p75) and were analyzed using a *t*-test or a nonparametric Mann–Whitney U-test (for two groups) or a Kruskal–Wallis test (for more than two groups). Categorical variables were expressed as numbers (%) and evaluated using a chi-square test or Fisher’s exact test, as appropriate. Correlations between two continuous variables were analyzed using Spearman correlation analysis. The predictive value of IL-35 for the prognosis of HBV-ACLF was assessed using area under receiver operating characteristic (AUROC) curves and Kaplan–Meier survival analysis. Differences were considered significant at two-tailed *p* < 0.05.

## 3. Results

### 3.1. Baseline Characteristics of Enrolled Participants

The demographic and clinical characteristics of all enrolled subjects are summarized in Table 1. Among the 69 patients with HBV-ACLF, the mean age was 48.3 years, with a significant male predominance (59/69, 85.5%). HBV reactivation was the most common trigger among all enrolled HBV-ACLF patients, so nucleoside analogs were prescribed for all patients. During the observation period, 34 patients died and 2 patients underwent living-donor liver transplantation during the observation period (ACLF-NS subgroup), while the remaining 33 patients recovered following medical treatment (ACLF-S subgroup). The non-survivors exhibited a higher proportion of liver cirrhosis than the survivors (72.2% vs. 42.4%, respectively). Additionally, compared to the survivors, the non-survivors showed significantly elevated levels of HBV-DNA, AST, TBIL, INR, PT, and lactate, while levels of PTA and AFP were reduced (all *p* < 0.05).

The complications and outcomes between the ACLF-S and ACLF-NS groups are shown in Table 2. Patients with HBV-ACLF were classified into three grades based on the frequency of organ failure(s): ACLF-1 (52.2%, 36/69), ACLF-2 (43.5%, 30/69), and ACLF-3 (4.3%, 3/69). The LT-free cumulative mortality rates of HBV-ACLF were 11.6% (8/69), 42% (29/69), and 49.3% (34/69) at 28, 90, and 180 days, respectively. The most common organ failure in patients with HBV-ACLF was liver failure (100%, 69/69), followed by coagulation failure (47.8%, 33/69), and cerebral failure (4.3%, 3/69). Overall, 78.3% of the patients (54/69) experienced at least one complication, with the most common being infection (60.9%, 42/69), ascites (46.4%, 32/69), and HE (17.3%,12/69), followed by renal dysfunction (5.8%, 4/69) and gastrointestinal bleeding (5.8%, 4/69). Infection (77.7% vs. 42.4%, *p* = 0.003), ascites (58.3% vs. 33.3%, *p* = 0.038), and HE (30.5% vs. 3%, *p* = 0.003) were significantly more frequent in the ACLF-NS group than in the ACLF-S group. The mortality of ACLF patients with three or more complications was as high as 90% (9/10). Moreover, the non-survivors exhibited higher MELD and COSSH-ACLF II scores than the survivors (*p* < 0.001).

### 3.2. The Plasma IL-35 Levels of All Participants

**Figure 1:**
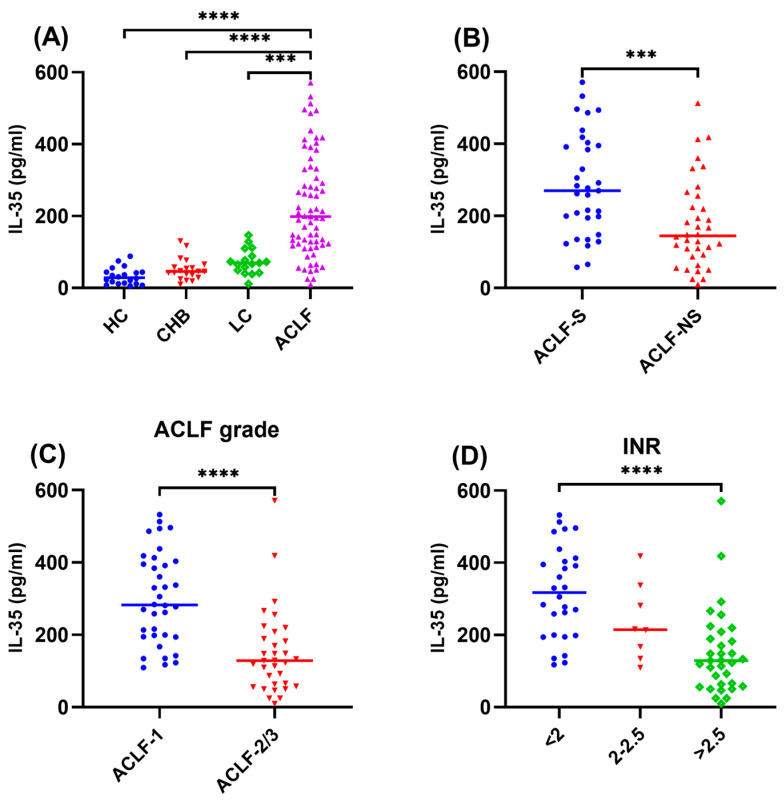
The baseline levels of plasma IL-35 were measured in enrolled participants. The levels of plasma IL-35 were compared in different disease stages (**A**), clinical outcomes (**B**), ACLF grades (**C**), and groups of HBV-ACLF patients divided by INR (<2, 2–2.5, >2.5) (**D**). *** *p* < 0.001, **** *p* < 0.0001. IL-35, interleukin-35; HBV, hepatitis B virus; ACLF, acute-on-chronic liver failure; INR, international normalized ratio.

As shown in Figure 1A, the baseline plasma IL-35 level in the HBV-ACLF group was 198.6 (120.9, 330.8) pg/mL, which was significantly higher than that in the LC [69.3 (46, 99.2) pg/mL, *p* < 0.001], CHB [45.9 (30.7, 63.5) pg/mL, *p* < 0.0001] and HC groups [28.6 (11.9, 44) pg/mL, *p* < 0.0001]. Additionally, the IL-35 levels were significantly higher in the ACLF-S group compared to the ACLF-NS group [267 (171.3, 399.3) pg/mL vs. 145 (88.1, 247.6) pg/mL, *p* = 0.001] (Figure 1B). The plasma IL-35 levels in ACLF-1 group were markedly higher than those in the ACLF-2/3 [282.5 (195.7, 401.2) pg/mL vs. 128.6 (60.5, 198.6) pg/mL, *p* < 0.0001] (Figure 1C). Plasma IL-35 levels were lower in the INR > 2.5 group compared to the INR < 2 group [128.6(60.5, 198.6) pg/mL vs. 317.7 (198.9, 410.4) pg/mL, *p* < 0.0001] (Figure 1D).

### 3.3. The Relationship Between Plasma IL-35 Levels and Complications in HBV-ACLF

**Figure 2:**
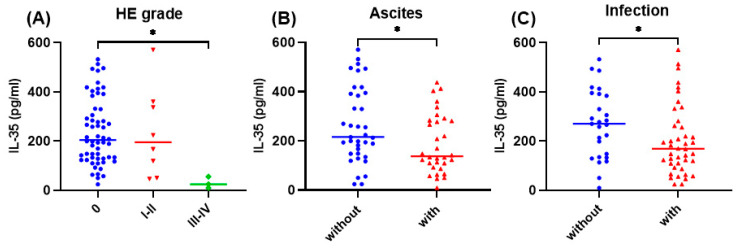
The plasma levels of IL-35 were compared in HBV-ACLF patients with different complications: HE (**A**), ascites (**B**), and infection (**C**). * *p* < 0.05. IL-35, interleukin-35; HBV, hepatitis B virus; ACLF, acute-on-chronic liver failure; HE, hepatic encephalopathy.

In HBV-ACLF patients with HE grade III-IV (Figure 2A), ascites (Figure 2B), and infection (Figure 2C), plasma IL-35 levels were significantly lower compared to those without such complications (all *p* < 0.05).

### 3.4. The Correlation of Plasma IL-35 Levels with Clinical Variables in HBV-ACLF

**Figure 3:**
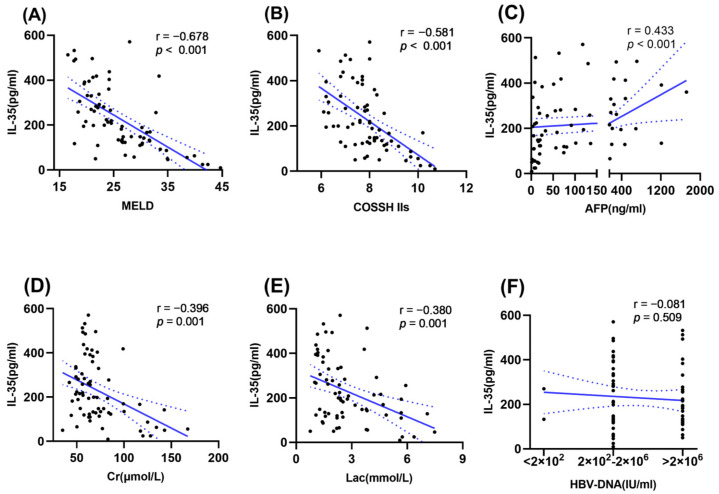
The correlation analysis between plasma IL-35 levels and MELD (**A**), COSSH IIs (**B**), AFP (**C**), Cr (**D**), lactate (**E**), and HBV-DNA (**F**) in HBV-ACLF. IL-35, interleukin-35; HBV, hepatitis B virus; ACLF, acute-on-chronic liver failure; MELD, model for end-stage liver disease; COSSH IIs, Chinese Group on the Study of Severe Hepatitis B ACLF II score; AFP, alpha fetal protein; Cr, creatinine.

To better understand the value of IL-35 in HBV-ACLF, we further analyzed the correlation between IL-35 and various clinical variables. As shown in Figure 3, the plasma IL-35 levels were negatively correlated with the levels of MELD (r = −0.678, *p* < 0.001), COSSH-ACLF IIs (r = −0.581, *p* < 0.001), Cr (r = −0.396, *p* = 0.001), and lactate (r = −0.380, *p* = 0.001). Conversely, IL-35 levels were positively associated with AFP levels (r = 0.433, *p* < 0.001). However, the plasma levels of IL-35 were not significantly correlated with HBV-DNA (r = −0.081, *p* = 0.509).

### 3.5. The Value of IL-35 in Predicting Outcomes of Patients with HBV-ACLF

**Figure 4:**
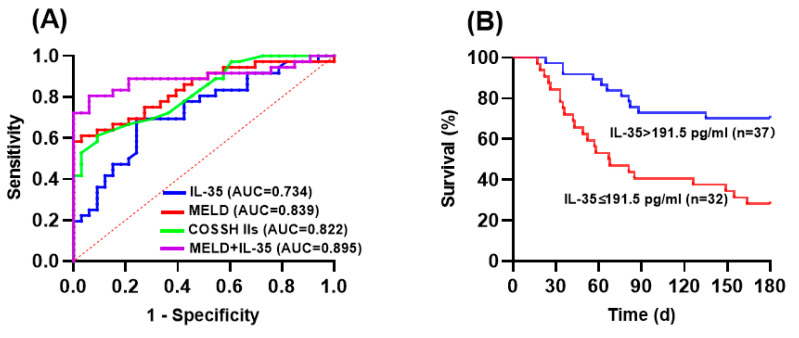
ROC curves of the baseline plasma IL-35 levels, MELD score, COSSH IIs, and MELD+IL-35 for predicting outcomes in HBV-ACLF patients (**A**). Kaplan–Meier survival curves of HBV-ACLF patients with different plasma IL-35 levels (**B**). IL-35, interleukin-35; HBV, hepatitis B virus; ACLF, acute-on-chronic liver failure; ROC, receiver operating characteristic; MELD, model for end-stage liver disease; COSSH IIs, Chinese Group on the Study of Severe Hepatitis B ACLF II score.

To investigate the relationship between plasma IL-35 levels and mortality in HBV-ACLF patients, we conducted an ROC analysis to compare the prognostic value of IL-35 levels with other scoring models. As shown in Figure 4A, the AUROC values were 0.734 (95%CI: 0.617–0.852, *p* = 0.001) for IL-35, 0.839 (95%CI: 0.746–0.933, *p* < 0.001) for MELD score, and 0.822 (95%CI: 0.727–0.918, *p* < 0.001) for COSSH IIs. The combination of plasma IL-35 and MELD score had the highest predictive efficiency for 180-day mortality, with an AUC of 0.895 (95% CI: 0.812–0.978, *p* < 0.001), a sensitivity of 80.6%, and a specificity of 93.9%.

Based on the Youden index, the optimal cut-off value of plasma IL-35 levels for outcome prediction was 191.5 pg/mL. Enrolled patients were further stratified into two groups according to their threshold value. The Kaplan–Meier analysis (Figure 4B) revealed that ACLF patients with plasma IL-35 levels ≤ 191.5 pg/mL had poorer survival times than those with plasma IL-35 levels > 191.5 pg/mL (*p* < 0.001).

## 4. Discussion

HBV-ACLF is a life-threatening disease characterized by systemic inflammation, rapid deterioration, and high short-term mortality. In our study, the prognosis of HBV-ACLF patients was poor, with nearly half (34/69, 49.3%) of the HBV-ACLF patients dying within 180 days of diagnosis, and two patients underwent living-donor liver transplantation. Therefore, early and accurate evaluation of the severity and prognosis of HBV-ACLF is critical for optimizing therapeutic decisions, preventing or delaying the progression, and reducing mortality in this population.

Previous reports have revealed that inflammatory response and immune imbalance triggered by HBV exacerbation drive the development and progression of HBV-ACLF [9,10,22]. IL-35, an anti-inflammatory and immunosuppressive cytokine of the IL-12 family, has been shown to hold diagnostic and therapeutic potential in several liver diseases, including chronic viral hepatitis, liver cirrhosis, and hepatocellular carcinoma (HCC). Previous studies have indicated that IL-35 is important for viral persistence, immune tolerance, and reducing liver inflammation during HBV infection [15,23]. Several studies have shown that IL-35 is highly expressed in blood and tumor tissue of HCC and promotes tumor metastasis, demonstrating that IL-35 derived from tumor cells also contributes to tumor progression [15,24]. Shi et al. reported that the mRNA and protein levels of IL-35 were significantly increased in patients with HBV-related liver cirrhosis, indicating that IL-35 is involved in the progression of cirrhosis [25]. Moreover, IL-35 might suppress the activation of the TGF-β1/Smad2/3 signaling pathway and inhibit the differentiation of Th17 cells in HBV-related liver cirrhosis patients, thereby suppressing inflammation, apoptosis, and fibrosis [26,27,28]. Zheng et al. demonstrated that IL-35 ameliorates liver injury by enhancing the secretion of IL-10 by Kupffer cells [29]. Koda et al. found that plasmacytoid dendritic cell (pDC)-derived IL-35 restricts liver damage by enhancing Treg activity in an autocrine manner [18]. Chen et al. found that serum IL-35 levels and Treg/Th17 ratios were higher in patients with prophase of liver failure who showed improvement than those who developed overt liver failure. Therefore, they inferred that lower levels of interleukin-35 in patients with prophase of liver failure are associated with progression to liver failure [19]. Taken together, the above data show that IL-35 can not only promote the chronicity of infection and the progression of hepatocellular carcinoma but also alleviate liver inflammation and inhibit liver fibrosis. Although various studies in the last decade have suggested that IL-35 may play a crucial role in the process of liver diseases through immunosuppressive regulation, its exact function, prognostic significance, and molecular mechanism still need further research.

In our present study, HBV-ACLF patients had higher IL-35 levels than the LC, CHB, and HC groups. This finding supports the notion that IL-35 is an anti-inflammatory cytokine secreted secondarily to inflammatory stimuli to limit excessive inflammation and immune-mediated liver injury [15,19]. To further examine the potential role of IL-35 in the prognosis of HBV-ACLF, patients were divided into the ACLF-S and ACLF-NS groups based on their outcomes. Interestingly, higher levels of plasma IL-35 were associated with a better prognosis. Moreover, lower levels of plasma IL-35 were correlated with disease severity and the occurrence of complications. Based on this finding, we speculated that IL-35 may be associated with the severity and outcomes of patients with HBV-ACLF.

Currently, the MELD score is widely used to evaluate the prognosis of ACLF patients [7]. It is commonly recognized that elevated MELD scores are associated with a poor prognosis. In our study, the MELD score was significantly elevated in the ACLF-NS group. Correlations between plasma IL-35 levels and clinical prognostic models (such as MELD and COSSH-ACLF IIs) were observed, indicating that IL-35 levels were associated with liver injury and disease progression. Furthermore, when combined with the MELD score, IL-35 showed the highest efficiency in predicting 180-day mortality, and the difference was statistically significant. The results of the Kaplan–Meier analysis indicated that the risk of mortality was correlated with lower levels of plasma IL-35 in patients with HBV-ACLF. These findings suggest that IL-35 is a reliable predictor and can improve predictive performance when combined with the MELD score.

The present study has several limitations. Firstly, plasma IL-35 levels were tested in a single-center cohort, and future multicenter studies with larger prospective cohorts should be conducted to validate our findings. Secondly, the predictors were collected at baseline without dynamic observation of disease progression. Additionally, the molecular mechanisms underlying the involvement of plasma IL-35 in HBV-ACLF progression remain unclear and warrant further investigation. Despite these limitations, the present prospective study is the first to demonstrate that IL-35 can serve as a promising predictor of mortality. Our findings provide a meaningful clue that enhances the diagnostic accuracy of the MELD score by incorporating IL-35 as a non-invasive biomarker, which has significant clinical implications.

## 5. Conclusions

The prognosis of HBV-ACLF remains unsatisfactory. Plasma IL-35 levels are elevated in patients with HBV-ACLF, and relatively higher levels are associated with a better prognosis. A combination of circulating IL-35 and MELD at the time of diagnosis appears to be more effective in predicting the mortality of HBV-ACLF. We suggest that IL-35 could be a promising and effective predictive marker for the prognosis of HBV-ACLF and may improve predictive performance when combined with the MELD score.

## Figures and Tables

**Table 1:** Clinical characteristics of all enrolled participants.

Characteristics	HBV-ACLF			LC (n = 17)	CHB (n = 20)	HCs (n = 20)	*p*-Value
	Total (n = 69)	Survivors (n = 33)	Non-Survivors (n = 36)				
Age (years)	48.3 ± 13.2	45.9 ± 13.3	50.5 ± 12.9	48.9 ± 10.2	40.8 ± 5.7	42.4 ± 10.3	0.153
Male (N, %)	59 (85.5%)	27 (81.8%)	32 (88.9%)	13 (76.5%)	12 (60%)	9 (45%)	0.502
HBV-DNA (IU/mL)							0.029
<2 × 10^2^	2 (2.9%)	2 (6.1%)	0	17 (100%)	20 (100%)	0	
2 × 10^2^–2 × 10^6^	35 (50.7%)	20 (60.6%)	15 (41.7%)	0	0	0	
>2 × 10^6^	32 (46.4%)	11 (33.3%)	21 (58.3%)	0	0	0	
Cirrhosis (n, %)	40 (58%)	14 (42.4%)	26 (72.2%)	17 (100%)	0	0	0.016
ALT (U/L)	299 (128.3, 556)	274 (115.5, 486)	331.5 (146, 819.8)	24 (15.7, 36)	19.2 (14.5, 34.7)	18.8 (13, 23.8)	0.364
AST (U/L)	202 (119.2, 390)	146 (98, 237.5)	250.7 (150.5, 422)	26 (21.8, 29.7)	20.8 (18, 24.9)	16.4 (13.7, 19)	0.007
AKP (U/L)	149.5 ± 39.4	151.9 ± 32.3	147.3 ± 45.2	84.1 ± 28	79 ± 18.8	69.1 ± 17.7	0.635
GGT (U/L)	77 (53.5, 130)	108 (57.5, 139)	75.5 (45.3, 108)	33 (17, 37)	23 (13.3, 35.75)	20 (12.3, 30.8)	0.87
TB (μmol/L)	397 (284.9, 488)	378 (260, 450.)	418.7 (343, 542.6)	18.4(11, 24.5)	11.8(10.3, 15.5)	12(9.7, 15.5)	0.049
Alb (g/L)	31.4 (28.4, 35.6)	32.5 (29.6, 36.4)	31.1 (28.1, 33.9)	40.6(39, 44.4)	46.9(43.2, 48.3)	47.8(44.8, 51)	0.124
Cr (μmol/L)	64 (56.1, 81.5)	62.5 (56.5, 71.6)	65.1 (53.2, 103.6)	80.2 (66.4, 87)	75.9 (58.3, 86.9)	63 (55.3, 74.1)	0.173
INR	2.5 (1.8, 3.5)	1.8 (1.6, 2.5)	3.3 (2.2, 4.2)	1.1 (1, 1.2)	1 (0.9, 1.1)	-	<0.001
PTA (%)	29.5 (22, 45.3)	44 (30, 49)	23 (18, 34)	79 (73.5, 90.5)	107 (91, 110.8)	-	<0.001
PT (s)	27.4 (20, 34.1)	20.3 (19.1, 27.3)	32.5 (24.7, 39.6)	14.8 (13,14.8)	13 (12.7,13.7)	-	<0.001
WBC (10^9^/L)	6.6 (4.9, 10.3)	6.4 (4.9, 7.9)	7.6 (4.8, 11.8)	3.8 (3.3, 5.1)	5.2 (4.1, 6.1)	5.6 (4.5, 6.9)	0.212
N (10^9^/L)	4.24 (3.1, 6.5)	4.15 (3, 5.3)	4.5 (3.1, 9.6)	2 (1.3,3.3)	3 (1.6,3.8)	2.8 (2.4, 3.7)	0.197
HGB (g/L)	125 (106.8, 136)	126 (107,139)	124 (106, 136)	141 (133, 149)	158.5 (133, 165)	144 (137, 148)	0.597
PLT (10^9^/L)	95.5 (66, 132.8)	95 (74, 154)	100 (57, 131)	98 (47, 118)	167 (132, 221)	239 (183–270)	0.990
CRP (mg/L)	7.4 (4.1, 12.5)	7.9 (5.2, 13.2)	7 (3.5,11.4)	-	-	-	0.322
Lac (mmol/L)	2.3 (1.5, 3.8)	1.9 (1.2,2.7)	2.5 (1.8,4.8)	-	-	-	0.016
Sdium(mmol/L)	133.1 ± 6.3	134 ± 6.2	132.4 ± 6.3	140 ± 1.7	139 ± 1.9	-	0.291
AFP (ng/mL)	57.8 (14.5, 172)	134 (53.4, 268)	19.8 (8.3, 67.6)	2.2 (1.4, 5.8)	3.2 (1.6, 5.5)	1.9 (1.4, 3.1)	<0.001

Data are presented as means ± SD, medians (p25, p75), or numbers (%). *p*-Value (<0.05) for comparisons between the ACLF-S and ACLF-NS groups (Student’s *t*-test, Mann–Whitney U test, Chi-square test, or Fisher’s exact test). HBV, hepatitis B virus; ACLF, acute-on-chronic liver failure; LC, liver cirrhosis; CHB, chronic hepatitis B; HCs, health controls; ALT, alanine aminotransferase; AST, aspartate aminotransferase; AKP, alkaline phosphatase; GGT, glutamyl transferase; TB, total bilirubin; Alb, albumin; Cr, creatinine; INR, international normalized ratio; PTA, prothrombin activity; PT, prothrombin time; WCC, white cell count; N, neutrophil; HGB, hemoglobin; PLT, platelet; CRP, C-reactive protein; Lac, lactate; AFP, alpha fetal protein.

**Table 2:** Comparison of complications and outcomes between HBV-ACLF survival group and non-survival group.

Characteristics	Total (n = 69)	Survivors (n = 33)	Non-Survivors (n = 36)	*p*-Value
Organ failure (n, %)				
Liver	69 (100%)	33 (100%)	36 (100%)	<0.001
Coagulation	33 (47.8%)	7 (21.2%)	26 (72.2%)	<0.001
Kidney	0	0	0	1.0
Cerebral	3 (4.3%)	0	3(8.3%)	0.240
Lung	0	0	0	1.0
Circulation	0	0	0	1.0
1.5 ≤ INR < 2.5 (n, %)	36 (47.8%)	26 (78.8%)	10 (27.8%)	<0.001
Renal dysfunction (n, %)	4 (5.8%)	0	4 (11.1%)	0.115
HE grade I or II (n, %)	9 (13%)	1 (3%)	8 (22.2%)	0.029
Ascites (n, %)	32 (46.4%)	11 (33.3%)	21 (58.3%)	0.038
Infection (n, %)	42 (60.9%)	14 (42.4%)	28 (77.7%)	0.003
Gastrointestinal bleeding (n, %)	4 (5.8%)	1 (3%)	3 (8.3%)	0.616
Number of complications (n, %)				<0.001
0	15 (21.7%)	15 (45.5%)	0	
1–2	44 (63.8%)	17 (51.5%)	27 (75%)	
≥3	10 (14.5%)	1 (3%)	9 (25%)	
HBV-ACLF(COSSH)				<0.001
ACLF grade 1	36 (52.2%)	26 (78.8%)	10 (27.8%)	
ACLF grade 2	30 (43.5%)	7 (21.2%)	23 (63.9%)	
ACLF grade 3	3 (4.3%)	0	3 (8.3%)	
Severity scores				
COSSH-ACLF IIs	7.9 ± 1.1	7.6 ± 0.8	8.5 ± 1.0	<0.001
MELDs	26.2 ± 6.5	22.3 ± 3.3	29.7 ± 6.7	<0.001
LT-free mortality				
28-day	8 (11.6%)	0	8 (22.2%)	<0.001
90-day	29 (42%)	0	29 (80.6%)	<0.001
180-day	34 (49.3%)	0	34 (94.4%)	<0.001

Data are presented as means ± SD or numbers (%). *p*-Value (<0.05) for comparisons between the ACLF-S and ACLF-NS groups (Student’s *t*-test, Chi-square test, or Fisher’s exact test). Two patients with ACLF underwent liver transplantation and were considered lost to follow-up for the mortality calculation. HBV, hepatitis B virus; ACLF, acute-on-chronic liver failure; INR, international normalized ratio; HE, hepatic encephalopathy; COSSH-ACLF IIs, Chinese Group on the Study of Severe Hepatitis B ACLF II score; MELD, model for end-stage liver disease; LT, liver transplantation.

## Data Availability

The data that support the findings of this study are available on request from the corresponding author. The data are not publicly available due to privacy or ethical restrictions.
